# Antigenic Mimicry in Paraneoplastic Immune Thrombocytopenia

**DOI:** 10.3389/fimmu.2019.00523

**Published:** 2019-03-22

**Authors:** Guillaume Vial, Etienne Rivière, Anne-Aurélie Raymond, Chloé James, Sylvaine Di-Tommaso, Nathalie Dugot-Senant, Jean-William Dupuy, Mokrane Yacoub, Marie Parrens, Fréderic Saltel, Jean-François Viallard

**Affiliations:** ^1^Internal Medicine and Infectious Diseases Department, Haut Lévêque Hospital, University Hospital Centre of Bordeaux, Pessac, France; ^2^INSERM, Biology of Cardiovascular Diseases, U1034, Pessac, France; ^3^Biology of Cardiovascular Diseases, U1034, University of Bordeaux, Pessac, France; ^4^INSERM U1053, Oncoprot, Bordeaux University, Bordeaux, France; ^5^Laboratory of Hematology, University Hospital Centre of Bordeaux, Pessac, France; ^6^Plateforme d'Histopathologie, TBM-Core US 005, Bordeaux, France; ^7^Centre de Génomique Fonctionnelle, Plateforme Protéome, Université de Bordeaux, Bordeaux, France; ^8^Pathology Department, Pellegrin Hospital, CHU Bordeaux, Bordeaux, France; ^9^Pathology Department, Haut Lévêque Hospital, University Hospital Centre of Bordeaux, Pessac, France

**Keywords:** antigenic mimicry, solid tumors, tumor antigens, immune thrombocytopenia, proteomics

## Abstract

The association of immune thrombocytopenia (ITP) with cancer has been reported, but the causality of tumor cells in paraneoplastic ITP pathogenesis and maintenance has never been established. We analyzed the unusual case of refractory ITP and coincident urothelial tumor of the kidney with circulating high titer anti-GPIIBIIIA autoantibodies. Intriguingly, after nephrectomy, the patient recovered fully and her anti-GPIIBIIIA autoantibodies disappeared. Proteomic and immunohistochemistry analyses revealed erratic GPIIB expression by the tumor cells, suggesting possible antigenic mimicry chronically stimulating the immune system and leading to this patient's refractory ITP. Such previously unreported findings provide proof-of-concept that requires further confirmation with the prospective study of a larger number of patients.

## Background

Immune thrombocytopenic purpura (ITP) is a rare disease associating increased autoimmune platelet destruction and decreased platelet production leading to potentially life-threatening bleeding. Its link with immune deficiency has been reported (1), but its link with cancer has rarely been described in adults (2–7). ITP's complex pathogenesis, involving autoreactive B and T cells, leads to antiplatelet autoantibody production targeting platelet components, i.e., glycoproteins; three of the latter, including GPIIBIIIA, could be detected by a specific assay (8), with anti-GPIIBIIIA antibodies found in 35 to 55% of ITP patients (9, 10). We describe a link between ITP platelets and GPIIB-expressing tumor cells that suggests antigenic mimicry between them.

## Materials and Methods

### Patient

Clinical data were obtained from the patient's medical records. The patient gave informed consent to publish this report and conduct further specific researches on her case, in accordance with the procedures of the Ethics Review Board of the Université de Bordeaux, Bordeaux (France).

### Detection of Antiplatelet Antibodies

Antiplatelet antibodies were detected in serum as previously described (8).

### T Cells Phenotyping

Circulating T cells were phenotyped as previously described (11), with specific searches for CD3^+^HLADR^+^, CD3^+^CD4^+^HLADR^+^, and CD3^+^CD8^+^HLADR^+^ T cells before and after nephrectomy.

### Platelet Purification

Platelet purification was adapted from a previously described protocol (12). Briefly, blood was collected in anticoagulant-citrate-dextrose-A (ACD-A) tubes (BD Biosciences, Le-Pont-de-Claix, France) and centrifuged to collect platelet-rich plasma. ACD-A and apyrase (Sigma Aldrich, Saint-Quentin Fallaviers, France) were added to prevent platelet activation. Platelets were centrifuged and resuspended in *N*-2-hydroxyethylpiperazine-*N*'-2-ethanesulfonic acid (HEPES) tyrode buffer containing glucose, apyrase and 0.5 μM EDTA. Leukocytes were removed with magnetic Dynabeads^®^ pan-mouse IgG (Life Technologies, St-Aubin, France) coupled to anti-CD45 primary antibody (BD Biosciences).

### Proteomic Analyses

Platelets were lysed in radioimmunoprecipitation assay (RIPA) buffer (Sigma) supplemented with protease-inhibitor cocktail (Roche, Mannheim, Germany). A 6-mm^2^ area of a 2.5-μm–thick, formalin-fixed, paraffin-embedded (FFPE), tumor-tissue section was preselected on a hematoxylin-and-eosin–stained slide and microdissected with a PALM type 4 (Zeiss) laser microdissector. Tumor proteins were extracted from the tumor and platelet proteomes with formalin cross-link reversion were subjected to mass-spectrometry (MS) analysis, as previously described (13). The Mascot 2.5 algorithm identified proteins with the Proteome Discoverer 1.4 Software (Thermo Fisher Scientific, Illkirch, France) used in batch mode to search the *Homo sapiens* database (71,663 entries, Reference Proteome Set, release 2017_06) from http://www.uniprot.org/ website, allowing 2 missed enzyme cleavages. MS and MS/MS mass tolerances were set to 10 ppm and 0.02 Da. Methionine oxidation, lysine acetylation, and asparagine and glutamine deamidations as dynamic modifications, and cysteine carbamidomethylation as a static modification were sought. Peptides were validated with Proline software (http://proline.profiproteomics.fr/). Only peptides with 1.0% false-discovery rate (FDR), calculated using the Mascot “decoy” option, and a pretty rank = 1 were retained. Proteins were identified with ≥2 specific peptides and FDR <1.0%.

### Immunohistochemistry Analyses

The 2.5-μm–thick tissues sections were dewaxed, rehydrated and antigens were retrieved in EnVision^TM^ FLEX Target Retrieval Solution Low pH solution (Dako-Agilent, Santa-Clara, CA, US), and immunolabeled in an automated autostainer (Dako-Agilent, Santa-Clara, CA, US) using standard reagents provided by the manufacturer. The sections were incubated with anti-CD41 rabbit polyclonal antibody (HPA031168; Sigma), diluted 1:50, for 60 min at room temperature, then incubated with anti-rabbit IgG conjugated horseradish peroxidase (EnVision Flex/HRP, Dako-Agilent) for 20 min for signal amplification, revealed by 3,3′-diaminobenzidine (Dako). The slides were counterstained with hematoxylin, dehydrated, and mounted. Each immunohistochemical run contained a negative control (buffer, no primary antibody). Sections were examined with a Nikon-Eclipse 501 microscope, and images were acquired using NIS-Elements F.

## Results and Discussion

**Figure 1:**
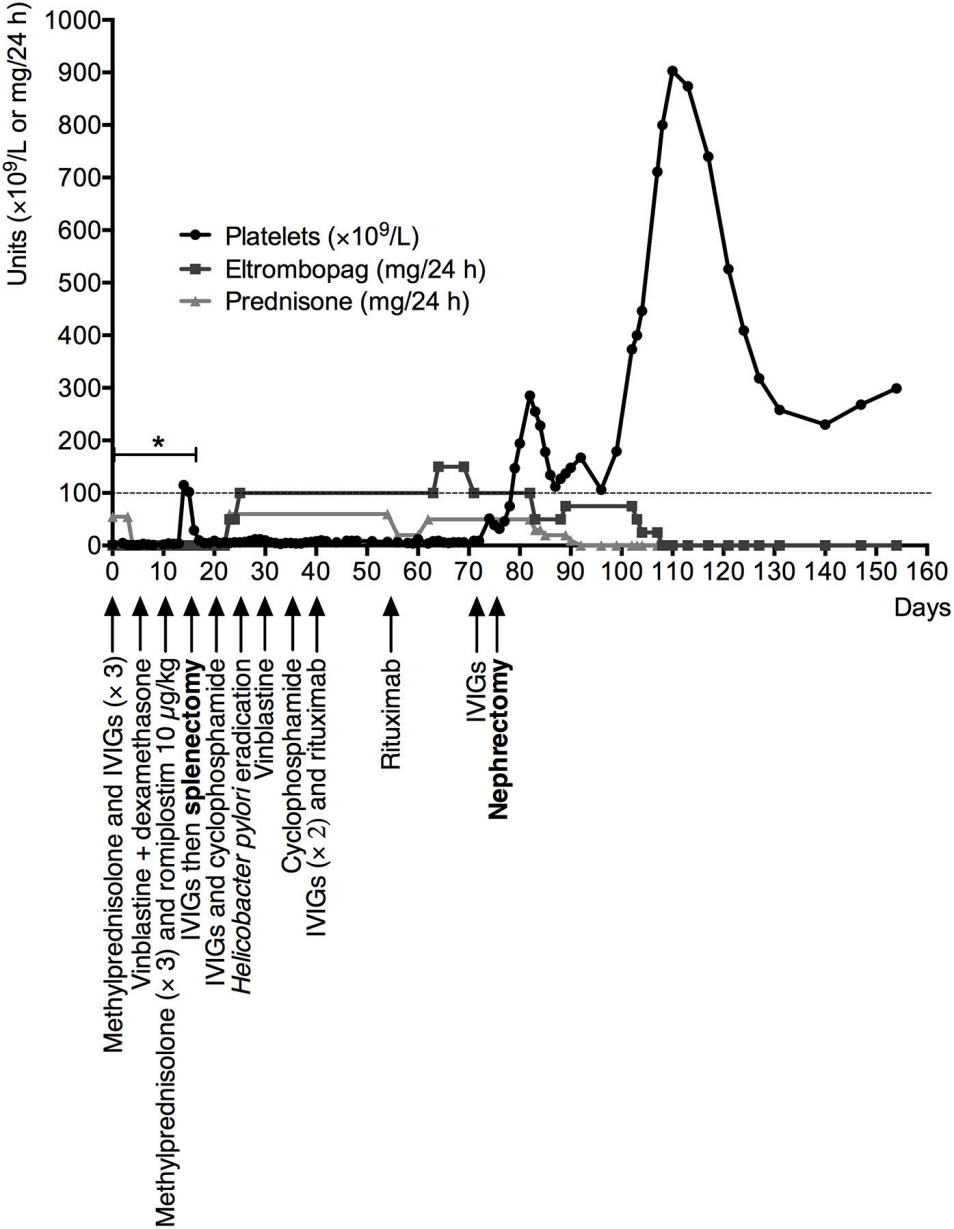
Patient's evolution under specific ITP treatments: platelet count (red line) as a function of days since ITP diagnosis and treatments administered (arrows), especially steroids (blue line), and eltrombopag (green line). *Intermittent bleeding was successive gastrointestinal hemorrhages, initially with and then without acute anemia, localized petechial or ecchymotic purpura and spontaneous hemorrhagic oral bullae. IVIGs, intravenous immunoglobulins.

A 70-year-old Caucasian woman was admitted for gastrointestinal bleeding and acute severe thrombocytopenia (2 × 10^9^/L), newly diagnosed as ITP (age >65 years, 2 purpura locations, hemorrhagic oral bullae and gastrointestinal bleeding with acute anemia) with a bleeding score (14) of 24. Her ITP failed to respond successively to steroids, immunoglobulins, vinblastine, romiplostim, emergent splenectomy, cyclophosphamide, rituximab, and eltrombopag. According to the international consensus (15) and after those therapeutic failures (Figure 1), refractory ITP was diagnosed. Her diagnostic work-up, including urine cytology and abdominal computed tomography, detected an intraepithelial papillary carcinoma of the right kidney and the urinary tract. At 1-year follow-up postnephrectomy, her thrombocytopenia was cured. Before nephrectomy, she had high-titer anti-GPIIBIIIA autoantibodies and elevated numbers of circulating activated CD3^+^CD8^+^HLADR^+^ cytotoxic T cells. Importantly, 9 days postnephrectomy, autoantibodies were no longer detectable in serum (titer at 2.05 before surgery vs. 0.67, 0.4, and 0.33, at 9, 54, and 166 days after surgery, respectively, with a positivity threshold at 1), and CD3^+^CD8^+^HLADR^+^ T cells returned to normal within 308 days. Given the complete ITP remission postsurgery, we hypothesized that the urothelial tumor had triggered and maintained antiplatelet autoimmunity through GPIIBIIIA antigenic mimicry mechanisms. To further explore this hypothesis, we compared the patient's platelets and tumor proteomes to look for common cell-surface proteins that could have triggered an autoimmune-response loop.

**Figure 2:**
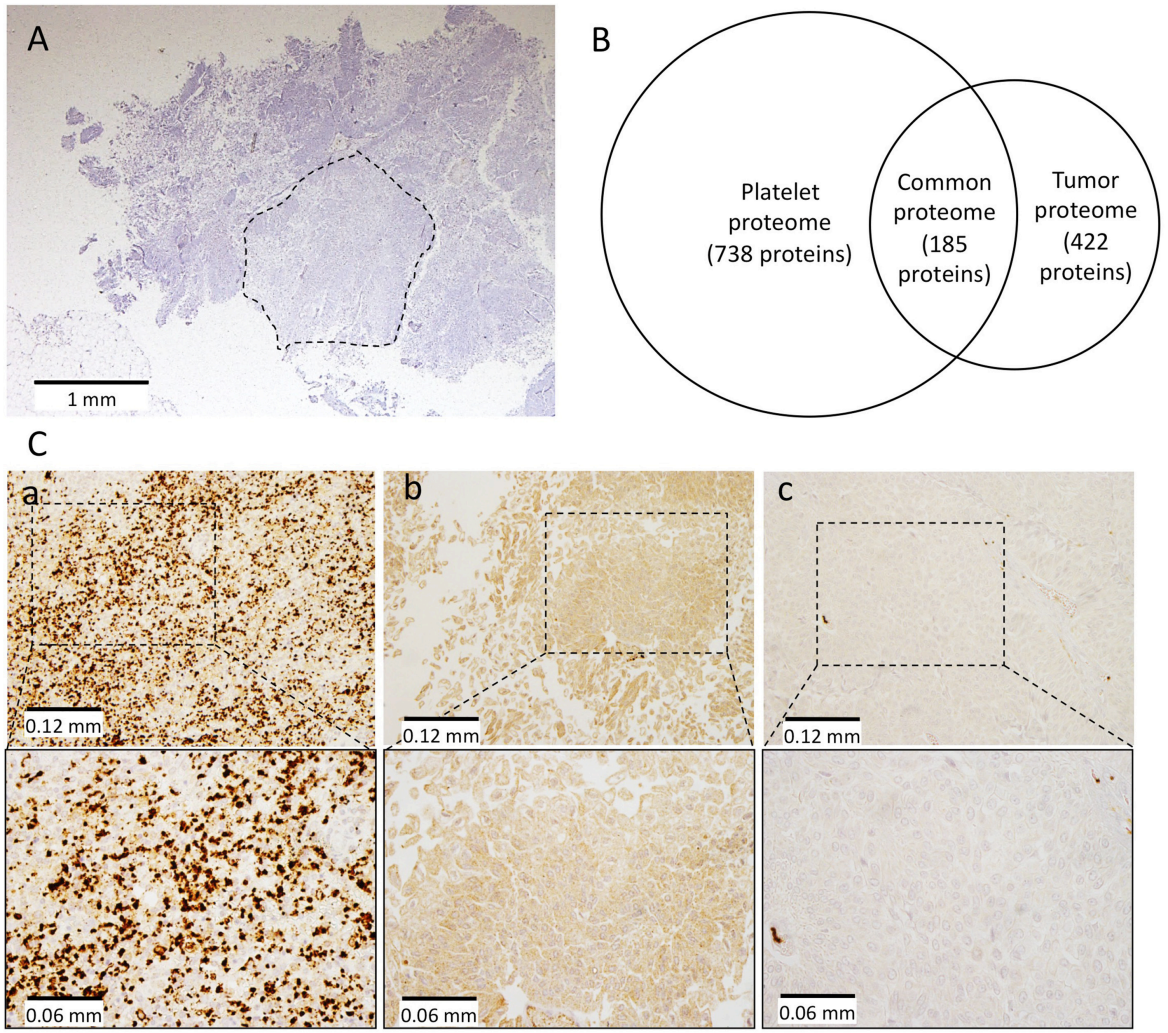
Proteomic and immunohistochemistry analyses on patient's platelets and tumor. Tissue was microdissected from a hematoxylin and eosin-stained FFPE section **(A)** (dotted lines show the microdissected area), tumor proteins were extracted and analyzed by MS and compared to the patient's platelet proteome. **(B)** Illustrates the overlapping of platelet and tumor proteomes. **(C)** Integrin α-IIb (also known as GPIIB or CD41) was detected immunohistochemically in the platelet-rich part of the patient's excised spleen (a) and tumor cells (b), but not in another patient's carcinoma cells (c) at low (top) and high magnification (bottom).

As postulated, integrin α-IIb (also known as GPIIB or CD41) was found among the 738 proteins identified in purified platelets (Figure 2B), and validated with 57 specific peptides. Surprisingly, integrin α-IIb was also found among the 422 proteins identified in the microdissected tumor (Figure 2A) and validated with 2 specific peptides, suggesting tumor aberrant α-IIb expression, which would have triggered anti-GPIIBIIIA autoantibody production. Another explanation could be platelet contamination of the tumor sample subjected to proteomic analyses. Therefore, we looked for other platelet-specific proteins among identified tumor proteins. Proteomic analyses revealed only a partial overlap of the patient's platelet and tumor proteins, meaning her tumor was probably platelet-free. Notably, many ubiquitous proteins were present in both samples but only few specific peptides, e.g., GPIIB. To determine whether tumor cells expressed GPIIB or GPIIB peptides were just platelet contamination, immunohistochemical labeling was done. Pertinently, no blood vessels were detected in the histological preparation: we observed bright punctiform labeling in the platelet-rich zone of the patient's removed spleen (Figure 2Ca). Anti-CD41 antibody (diluted 1:50) diffusely labeled our patient's tumor cells (Figure 2Cb) but not the carcinoma cells from an anti-GPIIBIIIA antibody-negative patient (Figure 2Cc). That diffuse labeling, only observed with high antibody concentration, could suggest weak antigen expression on the carcinoma cell surface.

To summarize, proteomic analyses and immunohistochemistry results support the hypothesis of aberrant tumor-cell expression of GPIIB. Other cell-surface proteins (e.g., integrins α_6_ and β_1_ or a disintegrin and metalloproteinase domain-containing protein-10 (ADAM10), were also identified in the tumor and platelets. Because we did not test their potential antigenic mimicry, the hypothetical combined implication of several proteins in ITP cannot be excluded.

Antigenic mimicry is powerful in breaking immune self-tolerance (16). In our case, a slight structural or conformational change in the fast growing tumor cells could have induced a point mutation in critical epitopes of GPIIB, although we have been unable to prove it. These changes might have circumvented the established tolerance, first against the mimicry epitope (tumor cells GPIIB), and later to the original epitope (platelets GPIIB) by additional epitope spreading. T cells hold an important role in the autoimmune loop induced by antigenic mimicry (17, 18), but B cells also by diversifying the autoimmune repertoire via intramolecular B cell epitope spreading (19).

A general defect in tolerance has been shown in ITP, mainly because of a decreased level and function of regulatory T cells as shown in some studies (20, 21). Interestingly, some patients experienced ITP remission while their platelet count rose under TPO mimetic treatment (22, 23), therefore suggesting that a platelet count rise could restore immune tolerance, possibly to GPIIB as well, however this is not specifically proven (24). Furthermore, a break in peripheral immune tolerance has been shown in ITP with an insufficient activity of indoleamine 2,3-dioxygenase leading to an increased survival of autoreactive T cells (25). Therefore, antigenic mimicry through GPIIB might have led to the activation and persistence of an anti-platelet autoimmune loop in our patient.

The spectacular efficacy of nephrectomy on the patient's refractory ITP raises some confounding biases. Mainly, our patient received many immunosuppressive agents before surgery, notably rituximab, whose estimated initial response onset ranges between 7 and 56 days (26). Nevertheless, the complicated management of the initial gastrointestinal bleeding attributable to profound thrombocytopenia led to therapeutic escalation according to best practices (27), especially early splenectomy. However, her platelet count rose only postnephrectomy, suggesting a postsurgical time correspondence with the complete ITP remission.

We described full recovery from paraneoplastic refractory ITP with detectable anti-GPIIBIIIA autoantibodies in serum and tumor-cell expression of GPIIB, cured by nephrectomy with autoantibody clearance and immune-activation termination. Such a case has not been reported previously and further confirmation of our findings is needed. Should they be confirmed, systematic computed-tomography screening for cancer in elderly patients with newly diagnosed ITP could be recommended.

## Author Contributions

ER, GV, A-AR, FS, MY, MP, and J-FV designed the research. ER purified platelets. MY and MP selected the areas of interest for proteomic analyses. J-WD, SD-T, and A-AR ran the proteomic analyses. ND-S did the immunohistochemical-labeling studies. ER, GV, A-AR, CJ, FS, and J-FV wrote the final manuscript. ER, GV, A-AR, CJ, FS, MY, and J-FV analyzed data. All authors approved the final manuscript.

### Conflict of Interest Statement

The authors declare that the research was conducted in the absence of any commercial or financial relationships that could be construed as a potential conflict of interest.
